# Supplementary material for: Assessment of eight insulin resistance surrogate indexes for predicting metabolic syndrome and hypertension in Thai law enforcement officers
Source: PeerJ. 2023 May 29;11:e15463. doi: 10.7717/peerj.15463 (PMC10234272; doi:10.7717/peerj.15463)
Supplement: Supplemental Information 1 [file peerj-11-15463-s001.docx]

**Supplementary Table S1** The ability of different insulin surrogate markers to predict the metabolic syndrome and hypertension in younger police officers (age < 48 years)

| **IR surrogate index** | **AUC (95% CI)** | **p-value** | **Sensitivity (%)** | **Specificity (%)** | **cut-off** | **Youden index** |
| --- | --- | --- | --- | --- | --- | --- |
| **To predict Metabolic syndrome** | |  |  |  |  |  |
| BMI | 0.754 (0.739-0.770) | <0.001 | 59.95 | 78.28 | 25.80 | 0.382 |
| WC | 0.770 (0.754-0.784) | <0.001 | 65.96 | 72.18 | 84.00 | 0.381 |
| TyG index | 0.880 (0.868-0.891) | <0.001 | 87.40 | 77.03 | 8.85 | 0.644 |
| TG/HDL-C | 0.871 (0.859-0.883) | <0.001 | 79.51 | 80.50 | 1.53 | 0.600 |
| TyG-BMI | 0.859 (0.846-0.872) | <0.001 | 80.92 | 75.79 | 221.57 | 0.567 |
| TyG-WC | 0.898 (0.886-0.908) | <0.001 | 82.21 | 81.05 | 756.37 | 0.633 |
| METS-IR | 0.880 (0.868-0.892) | <0.001 | 82.80 | 78.10 | 38.46 | 0.609 |
| LAP | 0.894 (0.883-0.905) | <0.001 | 85.75 | 78.33 | 33.68 | 0.641 |
| VAI | 0.880 (0.868-0.891) | <0.001 | 82.33 | 78.88 | 1.89 | 0.612 |
| AIP | 0.871 (0.859-0.883) | <0.001 | 79.51 | 80.45 | 0.18 | 0.600 |
| **To predict hypertension** | |  |  |  |  |  |
| BMI | 0.637 (0.620-0.654) | <0.001 | 54.87 | 66.29 | 24.80 | 0.212 |
| WC | 0.631 (0.613-0.648) | <0.001 | 61.28 | 56.75 | 82.00 | 0.180 |
| TyG index | 0.663 (0.646-0.680) | <0.001 | 58.04 | 65.82 | 8.79 | 0.239 |
| TG/HDL-C | 0.630 (0.612-0.647) | <0.001 | 50.94 | 68.66 | 1.42 | 0.196 |
| TyG-BMI | 0.673 (0.655-0.689) | <0.001 | 68.53 | 56.22 | 208.41 | 0.248 |
| TyG-WC | 0.675 (0.658-0.692) | <0.001 | 71.25 | 53.44 | 706.66 | 0.247 |
| METS-IR | 0.652 (0.635-0.669) | <0.001 | 64.30 | 57.88 | 36.05 | 0.222 |
| LAP | 0.656 (0.638-0.673) | <0.001 | 52.53 | 70.68 | 33.89 | 0.232 |
| VAI | 0.613 (0.596-0.631) | <0.001 | 54.72 | 62.86 | 1.66 | 0.176 |
| AIP | 0.630 (0.612-0.647) | <0.001 | 51.32 | 68.25 | 0.15 | 0.196 |

Younger age, age < 48 years; BMI, body mass index; WC, waist circumference; TyG index, triglyceride glucose index; TG/HDL-c, triglycerides/high-density lipoprotein cholesterol ratio; TyG-BMI, TyG index with body mass index; TyG-WC, TyG index with waist circumference; METS-IR, a metabolic score for insulin resistance; LAP, Lipid accumulation product; VAI, Visceral obesity index; AIP, atherogenic index of plasma
