# Supplementary material for: Assessment of eight insulin resistance surrogate indexes for predicting metabolic syndrome and hypertension in Thai law enforcement officers
Source: PeerJ. 2023 May 29;11:e15463. doi: 10.7717/peerj.15463 (PMC10234272; doi:10.7717/peerj.15463)
Supplement: Supplemental Information 2 [file peerj-11-15463-s002.docx]

**Supplementary Table S2** The ability of different insulin surrogate markers to predict metabolic syndrome and hypertension in older police officers

| **IR surrogate index** | **AUC (95% CI)** | **p-value** | **Sensitivity (%)** | **Specificity (%)** | **cut-off** | **Youden index** |
| --- | --- | --- | --- | --- | --- | --- |
| **To predict Metabolic syndrome** | |  |  |  |  |  |
| BMI | 0.713 (0.700-0.726) | <0.001 | 67.11 | 64.43 | 24.77 | 0.315 |
| WC | 0.724 (0.711-0.736) | <0.001 | 41.73 | 92.63 | 89.00 | 0.344 |
| TyG index | 0.881 (0.871-0.890) | <0.001 | 83.08 | 81.02 | 8.93 | 0.641 |
| TG/HDL-C | 0.861 (0.851-0.870) | <0.001 | 81.23 | 76.95 | 1.35 | 0.582 |
| TyG-BMI | 0.841 (0.830-0.851) | <0.001 | 78.31 | 75.11 | 222.62 | 0.534 |
| TyG-WC | 0.888 (0.879-0.897) | <0.001 | 81.87 | 79.68 | 760.92 | 0.616 |
| METS-IR | 0.863 (0.853-0.873) | <0.001 | 80.85 | 75.70 | 38.24 | 0.566 |
| LAP | 0.883 (0.874-0.892) | <0.001 | 81.36 | 80.32 | 34.62 | 0.617 |
| VAI | 0.868 (0.858-0.877) | <0.001 | 79.58 | 79.64 | 1.79 | 0.592 |
| AIP | 0.861 (0.851-0.870) | <0.001 | 81.23 | 76.86 | 0.13 | 0.581 |
| **To predict hypertension** | |  |  |  |  |  |
| BMI | 0.625 (0.611-0.638) | <0.001 | 60.98 | 58.15 | 24.31 | 0.191 |
| WC | 0.604 (0.590-0.618) | <0.001 | 51.79 | 64.05 | 84.00 | 0.158 |
| TyG index | 0.613 (0.599-0.627) | <0.001 | 57.59 | 60.29 | 8.77 | 0.179 |
| TG/HDL-C | 0.587 (0.573-0.601) | <0.001 | 57.00 | 56.76 | 1.14 | 0.138 |
| TyG-BMI | 0.648 (0.635-0.662) | <0.001 | 59.72 | 63.54 | 216.28 | 0.233 |
| TyG-WC | 0.637 (0.623-0.651) | <0.001 | 65.56 | 56.20 | 727.54 | 0.218 |
| METS-IR | 0.628 (0.615-0.642) | <0.001 | 51.75 | 67.21 | 38.13 | 0.190 |
| LAP | 0.624 (0.610-0.638) | <0.001 | 59.05 | 60.71 | 28.75 | 0.198 |
| VAI | 0.581 (0.567-0.595) | <0.001 | 61.58 | 50.95 | 1.34 | 0.125 |
| AIP | 0.588 (0.574-0.601) | <0.001 | 56.92 | 56.76 | 0.06 | 0.137 |

Older age, age ≥ 48 years; BMI, body mass index; WC, waist circumference; TyG index, triglyceride glucose index; TG/HDL-c, triglycerides/high-density lipoprotein cholesterol ratio; TyG-BMI, TyG index with body mass index; TyG-WC, TyG index with waist circumference; METS-IR, metabolic score for insulin resistance; LAP, Lipid accumulation product; VAI, Visceral obesity index; AIP, atherogenic index of plasma
