# Supplementary material for: Assessment of eight insulin resistance surrogate indexes for predicting metabolic syndrome and hypertension in Thai law enforcement officers
Source: PeerJ. 2023 May 29;11:e15463. doi: 10.7717/peerj.15463 (PMC10234272; doi:10.7717/peerj.15463)
Supplement: Supplemental Information 3 [file peerj-11-15463-s003.docx]

**Supplementary Table S3** The ability of different IR markers and combined IR markers to predict the metabolic syndrome

| **IR surrogate index** | **AUC (95% CI)** | ***p*-value*** | **Sensitivity (%)** | **Specificity (%)** | **cut-off** | **Youden index** |
| --- | --- | --- | --- | --- | --- | --- |
| BMI | 0.729 (0.719-0.739) | <0.001 | 68.86 | 65.05 | 24.76 | 0.339 |
| WC | 0.742 (0.732-0.751) | <0.001 | 41.76 | 93.13 | 89.00 | 0.349 |
| TyG index | 0.881 (0.873-0.888) | <0.001 | 85.38 | 78.14 | 8.88 | 0.635 |
| TG/HDL | 0.864 (0.856-0.872) | <0.001 | 84.68 | 73.71 | 1.29 | 0.584 |
| TyG-BMI | 0.848 (0.840-0.856) | <0.001 | 79.93 | 74.55 | 221.59 | 0.545 |
| TyG-WC | 0.892 (0.885-0.899) | <0.001 | 81.29 | 80.72 | 760.77 | 0.620 |
| METS-IR | 0.869 (0.862-0.877) | <0.001 | 80.75 | 77.32 | 38.45 | 0.581 |
| LAP | 0.887 (0.880-0.894) | <0.001 | 82.45 | 79.95 | 34.62 | 0.624 |
| VAI | 0.871 (0.863-0.878) | <0.001 | 81.50 | 77.87 | 1.78 | 0.594 |
| AIP | 0.864 (0.856-0.871) | <0.001 | 84.68 | 73.69 | 0.11 | 0.584 |
| TyG-BMI+TyG-WC | 0.892 (0.885-0.899) | <0.001 | 85.42 | 76.38 | 973.13 | 0.618 |
| TyG-BMI+TG/HDL | 0.853 (0.845-0.862) | <0.001 | 80.05 | 75.58 | 223.93 | 0.556 |
| TyG-BMI+LAP | 0.882 (0.874-0.890) | <0.001 | 83.60 | 77.24 | 257.09 | 0.608 |
| TyG-BMI+VAI | 0.855 (0.846-0.864) | <0.001 | 80.30 | 75.75 | 224.48 | 0.561 |
| TyG-BMI+AIP | 0.849 (0.840-0.858) | <0.001 | 80.05 | 74.61 | 221.83 | 0.547 |
| TyG-WC+TG/HDL | 0.893 (0.886-0.901) | <0.001 | 80.38 | 81.88 | 765.10 | 0.623 |
| TyG-WC+METS-IR | 0.895 (0.888-0.903) | <0.001 | 80.26 | 82.54 | 803.44 | 0.628 |
| TyG-WC+AIP | 0.892 (0.885-0.900) | <0.001 | 81.45 | 80.70 | 760.73 | 0.622 |

*Null hypothesis, AUC = 0.5; BMI, body mass index; WC, waist circumference; TyG index, triglyceride glucose index; TG/HDL-c, triglycerides/high-density lipoprotein cholesterol ratio; TyG-BMI, TyG index with body mass index; TyG-WC, TyG index with waist circumference; METS-IR, metabolic score for insulin resistance; LAP, Lipid accumulation product; VAI, Visceral obesity index; AIP, atherogenic index of plasma.
