# Supplementary material for: Assessment of eight insulin resistance surrogate indexes for predicting metabolic syndrome and hypertension in Thai law enforcement officers
Source: PeerJ. 2023 May 29;11:e15463. doi: 10.7717/peerj.15463 (PMC10234272; doi:10.7717/peerj.15463)
Supplement: Supplemental Information 4 [file peerj-11-15463-s004.docx]

**Supplementary Table S4** The ability of different IR markers and combined IR markers to predict the hypertension

| **IR surrogate index** | **AUC (95% CI)** | ***p*-value*** | **Sensitivity (%)** | **Specificity (%)** | **cut-off** | **Youden index** |
| --- | --- | --- | --- | --- | --- | --- |
| BMI | 0.630 (0.619-0.640) | <0.001 | 53.68 | 65.76 | 24.80 | 0.194 |
| WC | 0.618 (0.607-0.629) | <0.001 | 50.74 | 66.36 | 84.00 | 0.171 |
| TyG | 0.634 (0.624-0.645) | <0.001 | 58.07 | 62.15 | 8.77 | 0.202 |
| TG/HDL | 0.604 (0.593-0.614) | <0.001 | 57.14 | 58.63 | 1.16 | 0.158 |
| TyG-BMI | 0.659 (0.648-0.669) | <0.001 | 65.25 | 58.34 | 211.54 | 0.236 |
| TyG-WC | 0.655 (0.644-0.665) | <0.001 | 64.45 | 58.73 | 727.47 | 0.232 |
| METS-IR | 0.638 (0.627-0.649) | <0.001 | 62.55 | 57.41 | 36.19 | 0.200 |
| LAP | 0.636 (0.626-0.647) | <0.001 | 59.36 | 61.26 | 28.81 | 0.206 |
| VAI | 0.529 (0.581-0.603) | <0.001 | 62.78 | 51.18 | 1.34 | 0.140 |
| AIP | 0.604 (0.593-0.614) | <0.001 | 56.87 | 58.86 | 0.06 | 0.157 |
| TyG-BMI+TyG-WC | 0.661 (0.649-0.672) | <0.001 | 63.33 | 60.79 | 944.55 | 0.241 |
| TyG-BMI+TG/HDL | 0.659 (0.649-0.672) | <0.001 | 58.04 | 65.50 | 219.42 | 0.235 |
| TyG-BMI+LAP | 0.657 (0.645-0.669) | <0.001 | 64.97 | 58.79 | 240.45 | 0.238 |
| TyG-BMI+VAI | 0.659 (0.647-0.671) | <0.001 | 62.20 | 61.23 | 216.01 | 0.234 |
| TyG-BMI+AIP | 0.659 (0.647-0.671) | <0.001 | 65.42 | 58.19 | 211.50 | 0.236 |
| TyG-WC+TG/HDL | 0.655 (0.643-0.667) | <0.001 | 64.32 | 58.76 | 728.97 | 0.231 |
| TyG-WC+METS-IR | 0.656 (0.644-0.668) | <0.001 | 62.93 | 60.27 | 767.73 | 0.232 |
| TyG-WC+AIP | 0.655 (0.643-0.667) | <0.001 | 64.42 | 58.73 | 727.51 | 0.232 |

*Null hypothesis, AUC = 0.5; BMI, body mass index; WC, waist circumference; TyG index, triglyceride glucose index; TG/HDL-c, triglycerides/high-density lipoprotein cholesterol ratio; TyG-BMI, TyG index with body mass index; TyG-WC, TyG index with waist circumference; METS-IR, metabolic score for insulin resistance; LAP, Lipid accumulation product; VAI, Visceral obesity index; AIP, atherogenic index of plasma.
